# Deep learning-assisted diagnosis of large vessel occlusion in acute ischemic stroke based on four-dimensional computed tomography angiography

**DOI:** 10.3389/fnins.2024.1329718

**Published:** 2024-04-10

**Authors:** Yuling Peng, Jiayang Liu, Rui Yao, Jiajing Wu, Jing Li, Linquan Dai, Sirun Gu, Yunzhuo Yao, Yongmei Li, Shanxiong Chen, Jingjie Wang

**Affiliations:** ^1^Department of Radiology, The First Affiliated Hospital of Chongqing Medical University, Chongqing, China; ^2^College of Computer and Information Science, Southwest University, Chongqing, China

**Keywords:** large vessel occlusion, stroke, deep learning, four-dimensional computed tomography angiography, artificial neural network

## Abstract

**Purpose:**

To develop deep learning models based on four-dimensional computed tomography angiography (4D-CTA) images for automatic detection of large vessel occlusion (LVO) in the anterior circulation that cause acute ischemic stroke.

**Methods:**

This retrospective study included 104 LVO patients and 105 non-LVO patients for deep learning models development. Another 30 LVO patients and 31 non-LVO patients formed the time-independent validation set. Four phases of 4D-CTA (arterial phase P1, arterial–venous phase P2, venous phase P3 and late venous phase P4) were arranged and combined and two input methods was used: combined input and superimposed input. Totally 26 models were constructed using a modified HRNet network. Assessment metrics included the areas under the curve (AUC), accuracy, sensitivity, specificity and F1 score. Kappa analysis was performed to assess inter-rater agreement between the best model and radiologists of different seniority.

**Results:**

The P1 + P2 model (combined input) had the best diagnostic performance. In the internal validation set, the AUC was 0.975 (95%CI: 0.878–0.999), accuracy was 0.911, sensitivity was 0.889, specificity was 0.944, and the F1 score was 0.909. In the time-independent validation set, the model demonstrated consistently high performance with an AUC of 0.942 (95%CI: 0.851–0.986), accuracy of 0.902, sensitivity of 0.867, specificity of 0.935, and an F1 score of 0.901. The best model showed strong consistency with the diagnostic efficacy of three radiologists of different seniority (k = 0.84, 0.80, 0.70, respectively).

**Conclusion:**

The deep learning model, using combined arterial and arterial–venous phase, was highly effective in detecting LVO, alerting radiologists to speed up the diagnosis.

## Introduction

Large vessel occlusion (LVO) resulting in acute ischemic stroke (AIS) is the leading cause of severe death and disability (Malhotra et al., 2017). Timely treatment can rescue salvageable brain tissue, prevent increased neurological loss and improve the long-term prognosis of AIS patients. Endovascular therapy has been proven effective in AIS patients with LVO (Campbell et al., 2015; Goyal et al., 2016), but its effectiveness is highly time-dependent (Meretoja et al., 2017). Therefore, it is essential to accurately and quickly identify LVO.

CT angiography (CTA) is commonly used for detecting LVO (Almekhlafi et al., 2019; Mayer et al., 2020). Currently, four-dimensional computed tomography angiography (4D-CTA) is an emerging technique that combines the non-invasive feature of CTA with the dynamic acquisition of DSA (Kortman et al., 2015), producing a time-resolved cerebral vascular diagram of the brain vessels from the base of the skull to the vertex (Menon et al., 2013). It displays dynamic changes of blood flow in intracranial vessels and helps detect LVO more sensitively and accurately (Wagemans et al., 2017). With optimized stroke management, the number of CTA examinations for suspected AIS is increasing, as is the workload of radiologists, which may result in a delayed diagnosis of LVO. Thus, rapidly and accurately detecting LVO on numerous CTAs suspected of having cerebrovascular problems is an urgent task.

Deep learning (DL) is a subset of machine learning which is a category of methods of artificial intelligence. By iteratively adjusting the weight layers in a deep neural network structure to transform input information into multiple levels of abstraction, DL automatically learns discriminative features and representations from data (Shen et al., 2017; Chan et al., 2020). DL has proven to be particularly useful for medical image segmentation and reconstruction, as well as for disease diagnosis and prediction. Several studies have shown that DL models have comparable or better diagnostic capabilities than human radiologists (Choi et al., 2021; Fujima et al., 2021), particularly in neuroradiology (Zaharchuk et al., 2018; Dai et al., 2020; Narayana et al., 2020).

We hypothesized that DL algorithms can effectively learn the information provided by dynamic CTA to detect LVO, thereby accelerating LVO diagnosis and ensuring timely treatment for patients. The purpose of this study was to develop and validate DL algorithm for LVO detection based on 4D-CTA, and to compare its diagnostic performance with that of radiologists of varying levels of seniority.

## Materials and methods

### Study participants

This retrospective study was approved by the institutional review board and ethics committee of the First Affiliated Hospital of Chongqing Medical University. Patients newly diagnosed with LVO from July 2020 to November 2021 in the Department of Neurology of our hospital were recruited. The inclusion criteria were: (1) age ≥ 18 years old, (2) diagnosis of anterior circulation LVO, (3) the “one-stop-shop” 4D-CTA + CTP examination. Patients with incomplete clinical data and poor-quality CTA scans were excluded. We also collected age-sex-matched non-LVO patients with AIS symptoms who underwent AIS protocol imaging but were negative for vessel occlusion as a control group during the same period.

A total of 134 LVO patients and 136 non-LVO patients were enrolled. Among these, 104 LVO patients and 105 non-LVO patients (July 2020 to August 2021) were included to construct the training set for DL model training, testing and internal validation at a ratio of 6:2:2. To validate the generalizability of the DL model, 30 LVO patients and 31 non-LVO patients (September 2021 to November 2021) formed the time-independent validation set.

### Imaging protocols

4D-CTA images were obtained from a 320-row detector CT scanner (Aquilion ONE, Canon Medical Systems Corporation, Otawara, Japan). The scanning parameters were: 80 kV, 150–310 mA, coverage of 140–160 mm, 512 × 512 matrix, reconstruction with adaptive iterative dose reduction, 1.0 mm slice thickness, and 1.0 mm interval. The scanner provides whole-brain perfusion and dynamic vasculature information in one single examination with a single rotation of the gantry. The 4D-CTA acquisition protocol performs 19 volumetric scans by using a whole brain dynamic volume intermittent mode in a total of 60s. It used a tube current boost in the arterial enhancement peak sequence. The first volume, acquired at 310 mA, served as a mask. Subsequently, Three-volume scans at 150 mA, six-volume scans at 300 mA, and four-volume scans at 150 mA were performed sequentially per 2 s, constituting the arterial phase (11–36 s). The last five-volume scans were collected every 5 s at 150 mA for the venous phase (40–60 s). Iodine 400 (Iopamidol 400, Bracco Sine, Italy) was injected intravenously with the high-pressure syringe. The P3T technique of high-pressure syringe system (MEDRAD Stellant CT Injection System, Bayer Medical Care, Pittsburgh, United States) automatically calculate the amount and rate of contrast agents based on the patient’s gender, weight, height, and contrast agent concentration, providing a personalized contrast injection regimen.

All 19 volumetric scans were imported into a post-processing workstation (Vitrea, fX,1.0, Canon Medical Systems Corporation, Japan) and time-density curve (TDC) was generated by automatically labeling the inflow artery and outflow vein. According to the TDC, the point at which the arterial curve peaked was defined as the arterial phase (P1). The time point when the arterial curve and the venous curve intersected was defined as the arterial–venous phase (P2), the time of the venous curve to peak as the venous phase (P3), and the first time point when the venous curve entered a plateau was defined as the late venous phase (P4; Wang et al., 2022).

### Definition of LVO

LVO was defined as the presence of a contrast filling defect in specific segments, including the intracranial internal carotid artery (ICA) from the clinoid segment to its terminus, M1/M2 segments of the middle cerebral artery (MCA; Luijten et al., 2022). Occlusions in isolated extracranial ICA, A1/A2, M3/M4 and posterior circulation were not included. The ground truth of LVO was established by a senior neuroradiologist (Y.M.L. with 26 years of experience), considering the patient’s history and all available imaging data.

### Image preprocessing and augmentation

**Figure 1:**
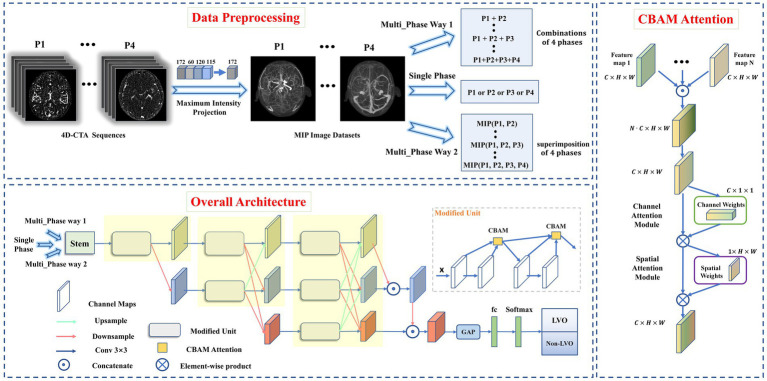
Data preprocessing pipeline (top), overall architecture of proposed model (left), and CBAM Attention architecture (right). Data preprocessing pipeline: The intracranial vascular MIP maps were obtained based on the four phases selected by 4D-CTA. The input format was divided into single-phase input and multi-phase input (where multi-phase input has two input methods: combination or superimposition). Overall architecture: the images obtained from MIP reconstruction were fed into the modified HRNet network to extract image features, then the extracted feature maps passed through the global average pooling layer and the fully connected layer. Finally, softmax was used to generate the predicted probability of each category as output. CBAM Attention improves the classification performance of the network by weighting the extracted features in space and channels.

The dataset construction process was shown in Figure 1. Bone removal data for the four phases (P1, P2, P3, and P4) were obtained by subtracting the images of the four defined phases from the mask images. Intracranial vascular MIP map for each phase was then obtained using the maximum intensity projection (MIP) reconstruction. The MIP algorithm was implemented by Python (3.8.0), mainly using Opencv library (4.5.3) and PIL library (8.0.1).

**Figure 2:**
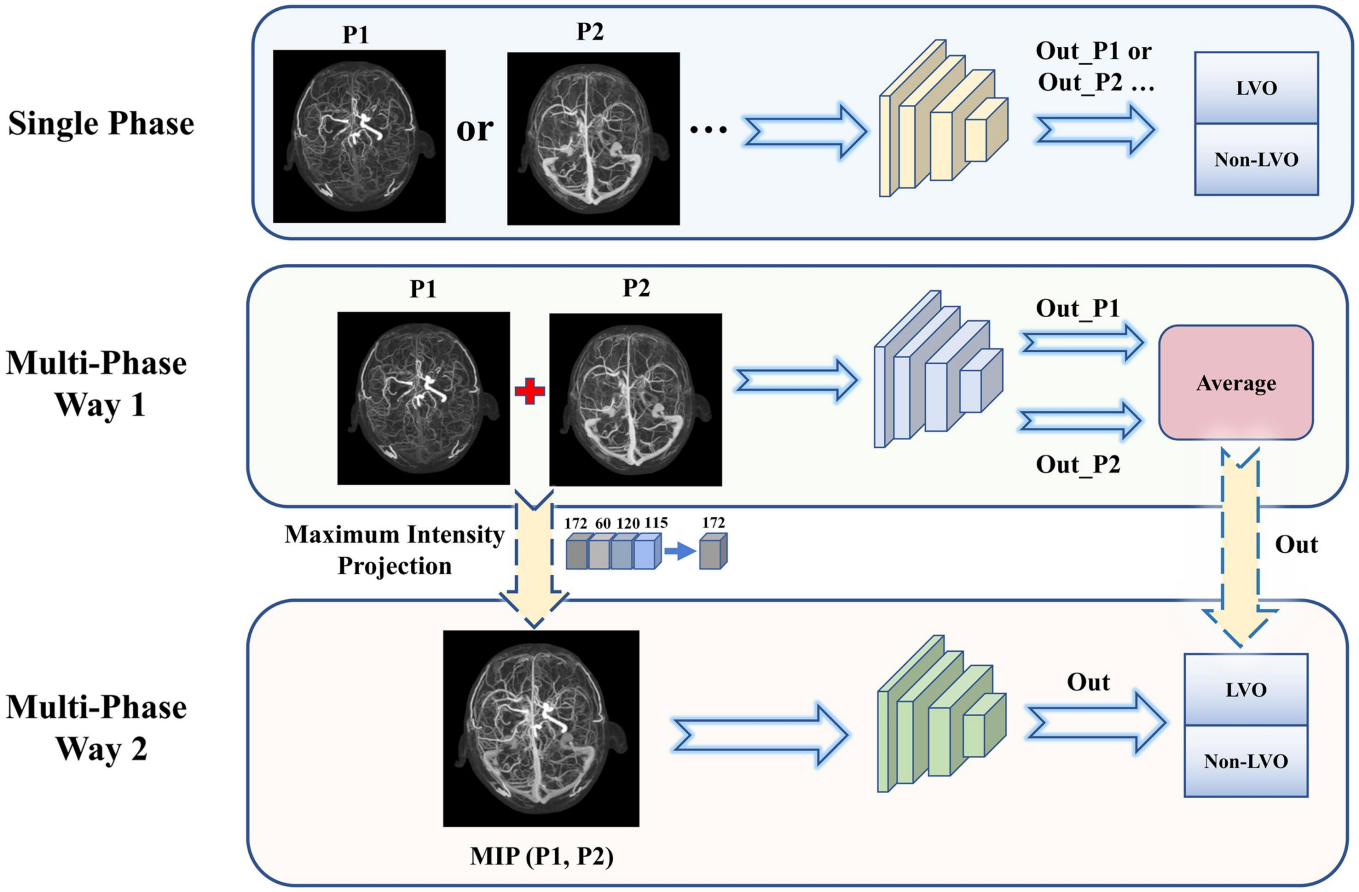
Three types of input to the model. Single phase (top): Each phase was input to the network model individually and the classification results were obtained. Multi-phase Way 1 (middle): Combination of multiple phase images for input. The output predicted probabilities were averaged to obtain the final model results. Multi-phase Way 2 (bottom): Superimposition of multiple phase images for input. The MIP images from multiple phases were again subjected to MIP processing to obtain a single superimposed image, which was input to obtain the final model classification results.

A total of 26 DL models were developed. Initially, four models were created separately for each phase (P1, P2, P3, and P4). Then, 11 combinations were formed based on the different combinations of four time phases. And each was trained separately using two different input methods. Input method 1—combination of different phase MIP maps, including P1 + P2, P1 + P3, P1 + P4, P2 + P3, P2 + P4, P3 + P4, P1 + P2 + P3, P1 + P2 + P4, P1 + P3 + P4, P2 + P3 + P4, and P1 + P2 + P3 + P4. Input method 2—superimposition of different phase MIP maps (i.e., re-projection of MIP maps from multiple phases into one using MIP reconstruction), including MIP (P1, P2), MIP (P1, P3), MIP (P1, P4), MIP (P2, P3), MIP (P2, P4), MIP (P3, P4), MIP (P1, P2, P3), MIP (P1, P2, P4), MIP (P1, P3, P4), MIP (P2, P3, P4), MIP (P1, P2, P3, P4). Refer to Figure 2 for details.

Conventional data enhancement techniques such as online rotation, flipping, cropping, and luminance changes were applied to enhance the learning representation of the model. These operations were implemented in Python (version 3.8.0).

### DL model development

We used the HRNet network to build DL model for LVO detection due to its superiority in image classification and segmentation (Jansen et al., 2018; Smith et al., 2018). To improve its performance, we made three modifications to the original HRNet network (Figure 1), It was mainly reflected in: (1) reduced the network structure from 4 to 3 stages (each yellow area represents one stage), (2) improved the successive convolution operations in each stage (modified to Modified Unit), (3) introduced channel and spatial attention mechanisms to the Modified Unit to enhance the network’s learning ability. In short, the network workflow involved feeding MIP-reconstructed images into the modified HRNet network to extract image features, then passed the feature maps through the global average pooling layer and fully connected layer, and finally generated the predicted probability of each category as output using softmax.

All models were implemented on the PyTorch library with NVIDIA GPU (GeForce GTX 2070 SUPER). The input image size was set to 256 × 256, and the batch size was set as 16. ADAM optimizer was used with an initial learning rate of 0.0001. The momentum and training epoch was set to 0.9 and 200, respectively. The warm-up strategy was applied in the first five epochs, and the learning rate was decayed at the 80th and 150th epochs with a decay ratio of 0.1.

### Radiologist performance

To evaluate the proposed DL model’s clinical applicability, we compared the diagnostic performance of the best model with that of radiologists with varying expertise. Three radiologists, Reader 1 (J.L with 8 years of experience), Reader 2 (L.Q.D with 5 years of experience), and Reader 3, a trainee radiologist (S.R.G, a trainee radiologist with 1 year of experience), identified LVO or non-LVO from 4D-CTA images of the temporal validation set. They were blinded to clinical information, radiologic reports, and other examination findings.

### Statistical analysis

The normality of data distribution was tested, with continuous variables reported as mean ± standard deviations (SD) or medians and interquartile ranges (IQRs), and categorical variables reported as proportions. Continuous variables were analyzed using Student’s t-test or Mann–Whitney U-test. The performance of the proposed DL models was evaluated by calculating the areas under the curve (AUC) using receiver operating characteristic curve (ROC) analysis for the training and temporal validation sets. We also compared performance using accuracy, sensitivity, specificity, positive predictive value (PPV), negative predictive value (NPV), and F1 score. Differences in ROC among the DL models were evaluated using the Delong test. Inter-rater agreement between the best model and each reader was assessed using the kappa analysis. Statistical significance was considered at *p* < 0.05. Statistical analysis was performed using SPSS (version 26.0, IBM, New York) and MedCalc software (version 20.115, https://www.medcalc.org/).

## Results

### Patient demographic data, clinical characteristics

Table 1 summarized the demographic characteristics of 270 subjects, including 134 LVO patients (83 males and 51 females) and 136 non-LVO patients (83 males and 53 females). The training set comprised 104 LVO patients (mean age, 65.88 ± 12.82 years; 67 men) and 105 non-LVO patients (mean age, 62.85 ± 10.77 years; 64 men). Of these, the LVO subgroup contained 63 M1 (60.58%), 19 M2 (18.27%), 14 ICA (13.46%), and 8 tandem (7.69%). The temporal validation set consisted of 30 LVO patients (mean age, 69.50 ± 9.89 years; 16 men) and 31 non-LVO patients (mean age, 65.51 ± 12.57 years; 19 men), with 19 M1 (63.33%), 6 M2 (20%), 2 ICA (6.67%), and 3 tandem (10%) in the LVO subgroup. Both sets showed that LVO patients had higher NIHSS median scores than non-LVO patients [9 (2–15) vs. 2 (1–4), *p* < 0.001; 11 (2–18) vs. 0 (0–1), *p* < 0.001]. In the overall cohort, a NIHSS score cut-off of 6.5 was identified as the optimal threshold for detecting LVO, yielding an AUC of 0.853 (95% CI 0.804–0.903). This NIHSS cut-off demonstrated a sensitivity of 69% and specificity of 96%.

**Table 1:** Demographics in the training and temporal validation cohort.

Characteristic	Training set (*n* = 209)			Temporal validation set (*n* = 61)		
	LVO (*n* = 104)	Non-LVO (*n* = 105)	*p*	LVO (*n* = 30)	Non-LVO (*n* = 31)	*p*
Age, mean (SD), years	65.88 ± 12.82	62.85 ± 10.77	0.066	69.50 ± 9.89	65.51 ± 12.57	0.175
Male, no. (%)	67 (64.42%)	64 (60.95%)	0.600	16	19	0.530
NIHSS score, median (IQR)	9 (2–15)	2 (1–4)	**<0.001**	11 (2–18)	0 (0–1)	**<0.001**
Target occlusion location, no. (%)						
ICA	14 (13.46%)	-	-	2 (6.67%)	-	-
M1 MCA	63 (60.58%)	-	-	19 (63.33%)	-	-
M2 MCA	19 (18.27%)	-	-	6 (20%)	-	-
ICA and MCA	8 (7.69%)	-	-	3 (10%)	-	-

The bold values represents significant differences in intergroup comparisons, with *p* < 0.05.

### Performance of the DL models

The DL models developed in four separate phases had AUCs above 0.700 (Table 2). Of these, model P1 and P2 showed superior performance with AUCs above 0.800. The DL model trained on P1 alone achieved an AUC of 0.844 (95%CI: 0.704–0.935), with accuracy of 0.867, sensitivity of 0.963, specificity of 0.722 and F1 score of 0.855. The DL model trained on P2 alone achieved an AUC of 0.897 (95%CI: 0.770–0.968), slightly higher than P1, with accuracy of 0.844, sensitivity of 0.889, specificity of 0.778 and F1 score of 0.836.

**Table 2:** The performance of the DL model based on four separate phases.

Single phase		AUC (95%CI)	Accuracy	Sensitive	Specificity	PPV	NPV	F1-score
P1	Internal validation set	0.844 (0.704–0.935)	0.867	0.963	0.722	0.929	0.839	0.855
	Temporal validation set	0.843 (0.727–0.924)	0.803	0.767	0.839	0.788	0.821	0.803
P2	Internal validation set	0.897 (0.770–0.968)	0.844	0.889	0.778	0.824	0.857	0.836
	Temporal validation set	0.838 (0.721–0.920)	0.803	0.967	0.645	0.952	0.725	0.799
P3	Internal validation set	0.770 (0.620–0.882)	0.756	0.778	0.722	0.684	0.808	0.748
	Temporal validation set	0.703 (0.573–0.813)	0.689	0.667	0.710	0.688	0.690	0.688
P4	Internal validation set	0.790 (0.643–0.897)	0.778	0.704	0.889	0.667	0.905	0.777
	Temporal validation set	0.555 (0.422–0.682)	0.590	0.900	0.290	0.750	0.551	0.551

The results of DL models using combinations of MIP maps from different phases as input way were shown in Table 3. All models had AUCs above 0.800 for the Internal validation set. Among them, the P1 + P2 model had superior diagnostic performance with an increased AUC of 0.975 (95%CI: 0.878–0.999) compared to model P1, and achieved an accuracy of 0.911, sensitivity of 0.889, specificity of 0.944, and an F1 score of 0.909. Consistently high diagnostic efficacy for the P1 + P2 model was observed in the temporal validation set, with an AUC of 0.942 (95% CI: 0.851–0.986), accuracy of 0.902, sensitivity of 0.867 and specificity of 0.935, and F1 score of 0.901. This was followed by the P1 + P2 + P3 model with an AUC of 0.916 (95%CI: 0.794–0.978), accuracy of 0.867, sensitivity of 0.852, specificity of 0.889, and F1 score of 0.863. However, the Delong test showed that the improvement was not statistically significant (Supplementary Table S1).

**Table 3:** The performance metrics for DL models with combinations of MIP maps at different stages as input way.

Combination of different phase		AUC (95%CI)	Accuracy	Sensitive	Specificity	PPV	NPV	F1-score
P1 + P2	Internal validation set	0.975 (0.878–0.999)	0.911	0.889	0.944	0.850	0.960	0.909
	Temporal validation set	0.942 (0.851–0.986)	0.902	0.867	0.935	0.879	0.929	0.901
P1 + P3	Internal validation set	0.842 (0.702–0.933)	0.822	0.852	0.778	0.778	0.852	0.815
	Temporal validation set	0.794 (0.671–0.887)	0.754	0.867	0.645	0.833	0.703	0.752
P1 + P4	Internal validation set	0.877 (0.744–0.956)	0.800	0.778	0.833	0.714	0.875	0.796
	Temporal validation set	0.905 (0.803–0.965)	0.770	0.900	0.645	0.870	0.711	0.767
P2 + P3	Internal validation set	0.823 (0.680–0.921)	0.778	0.815	0.722	0.722	0.815	0.769
	Temporal validation set	0.755 (0.628–0.856)	0.738	0.767	0.710	0.759	0.719	0.738
P2 + P4	Internal validation set	0.881 (0.749–9.058)	0.800	0.889	0.667	0.800	0.800	0.785
	Temporal validation set	0.787 (0.663–0.882)	0.705	0.800	0.613	0.760	0.667	0.703
P3 + P4	Internal validation set	0.809 (0.664–0.910)	0.733	0.704	0.778	0.636	0.826	0.730
	Temporal validation set	0.753 (0.626–0.854)	0.672	0.833	0.516	0.762	0.625	0.665
P1 + P2 + P3	Internal validation set	0.916 (0.794–0.978)	0.867	0.852	0.889	0.800	0.920	0.863
	Temporal validation set	0.926 (0.829–0.977)	0.820	0.867	0.774	0.857	0.788	0.819
P1 + P2 + P4	Internal validation set	0.856 (0.719–0.943)	0.822	0.963	0.611	0.917	0.788	0.800
	Temporal validation set	0.812 (0.691–0.900)	0.738	0.967	0.516	0.941	0.659	0.725
P1 + P3 + P4	Internal validation set	0.805 (0.659–0.907)	0.733	0.741	0.722	0.650	0.800	0.727
	Temporal validation set	0.827 (0.708–0.912)	0.672	0.833	0.516	0.762	0.625	0.665
P2 + P3 + P4	Internal validation set	0.819 (0.676–0.918)	0.800	0.778	0.833	0.714	0.875	0.796
	Temporal validation set	0.823 (0.704–0.909)	0.705	0.967	0.452	0.933	0.630	0.686
P1 + P2 + P3 + P4	Internal validation set	0.897 (0.770–0.968)	0.756	0.815	0.667	0.706	0.786	0.743
	Temporal validation set	0.705 (0.575–0.815)	0.656	0.867	0.452	0.778	0.605	0.642

**Figure 3:**
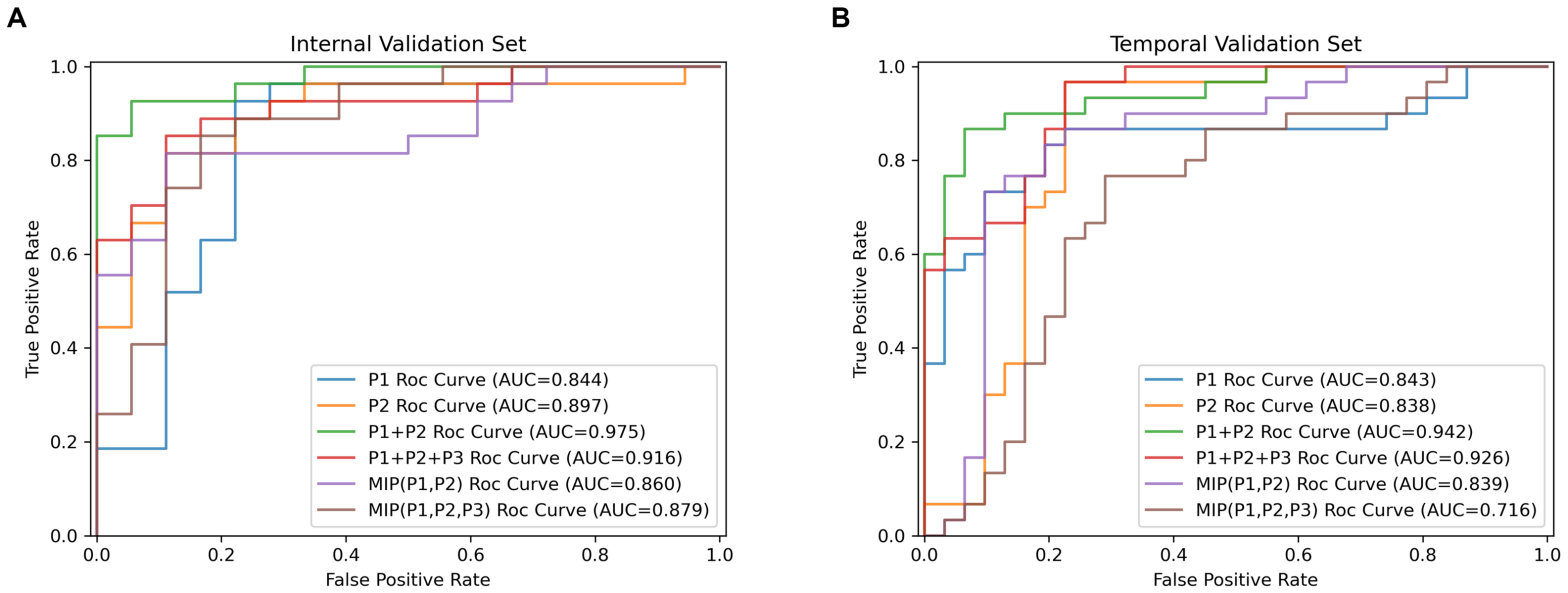
The receiver operating characteristic curves results (ROC) for the top two high-performing DL models which were, respectively, built by single-phase input, multi-phases combined input and multi-phases superimposed input way. **(A)** Results of ROC analysis on the internal validation cohort. **(B)** Results of ROC analysis on the temporal validation cohort.

Table 4 showed results of DL models using superimposition of MIP maps from different phases as the input method. The average AUC was 0.798, significantly lower than the mean AUC of 0.863 for the combination input models. The DL models trained on MIP (P1, P2, P3) and MIP (P1, P2) showed relatively good efficacy in identifying LVO, with AUC of 0.879 (95%CI: 0.747–0.957) and 0.860 (95%CI: 0.724–0.945), respectively. However, both models were less effective than the best model P1 + P2. ROC results for the top two high-performing DL models with different input methods were shown in Figure 3. Overall, we constructed 26 models, and the DL model trained on P1 + P2 using the combined input approach had the highest diagnostic efficacy, surpassing the models composed by the mono-phase and superimposed input approaches.

**Table 4:** The performance of DL models using superimposition of MIP maps from different phases as input way.

Superimposition of different phases		AUC (95%CI)	Accuracy	Sensitive	Specificity	PPV	NPV	F1-score
MIP (P1, P2)	Internal validation set	0.860 (0.724–0.945)	0.917	0.815	0.889	0.762	0.844	0.863
	Temporal validation set	0.839 (0.722–0.920)	0.781	0.833	0.774	0.828	0.803	0.806
MIP (P1, P3)	Internal validation set	0.815 (0.671–0.925)	0.793	0.852	0.667	0.750	0.778	0.821
	Temporal validation set	0.694 (0.562–0.805)	0.704	0.633	0.742	0.676	0.689	0.667
MIP (P1, P4)	Internal validation set	0.815 (0.671–0.925)	0.750	1.000	0.500	1.000	0.800	0.857
	Temporal validation set	0.709 (0.578–0.818)	0.714	0.500	0.806	0.625	0.656	0.588
MIP (P2, P3)	Internal validation set	0.842 (0.702–0.933)	0.875	0.778	0.833	0.714	0.800	0.824
	Temporal validation set	0.762 (0.636–0.862)	0.842	0.533	0.903	0.667	0.721	0.653
MIP (P2, P4)	Internal validation set	0.691 (0.536–0.820)	0.741	0.741	0.611	0.611	0.689	0.741
	Temporal validation set	0.629 (0.496–0.749)	0.520	0.867	0.226	0.636	0.541	0.650
MIP (P3, P4)	Internal validation set	0.753 (0.602–0.869)	0.933	0.519	0.944	0.567	0.689	0.667
	Temporal validation set	0.644 (0.511–0.763)	0.595	0.833	0.452	0.737	0.639	0.694
MIP (P1, P2, P3)	Internal validation set	0.879 (0.747–0.957)	0.880	0.815	0.833	0.750	0.822	0.846
	Temporal validation set	0.716 (0.586–0.824)	0.650	0.867	0.548	0.810	0.705	0.743
MIP (P1, P2, P4)	Internal validation set	0.805 (0.659–0.907)	0.905	0.704	0.889	0.667	0.778	0.792
	Temporal validation set	0.782 (0.657–0.877)	0.692	0.900	0.613	0.864	0.754	0.783
MIP (P1, P3, P4)	Internal validation set	0.720 (0.566–0.844)	0.778	0.778	0.667	0.667	0.733	0.778
	Temporal validation set	0.781 (0.656–0.876)	0.714	0.833	0.677	0.808	0.754	0.769
MIP (P2, P3, P4)	Internal validation set	0.784 (0.636–0.893)	0.870	0.741	0.833	0.682	0.778	0.800
	Temporal validation set	0.755 (0.628–0.856)	0.683	0.933	0.581	0.900	0.754	0.789
MIP (P1, P2, P3, P4)	Internal validation set	0.813 (0.669–0.913)	0.875	0.778	0.833	0.714	0.800	0.824
	Temporal validation set	0.675 (0.543–0.790)	0.634	0.867	0.516	0.800	0.689	0.732

### Discrimination performance of the best model and radiologists with different levels of expertise

**Figure 4:**
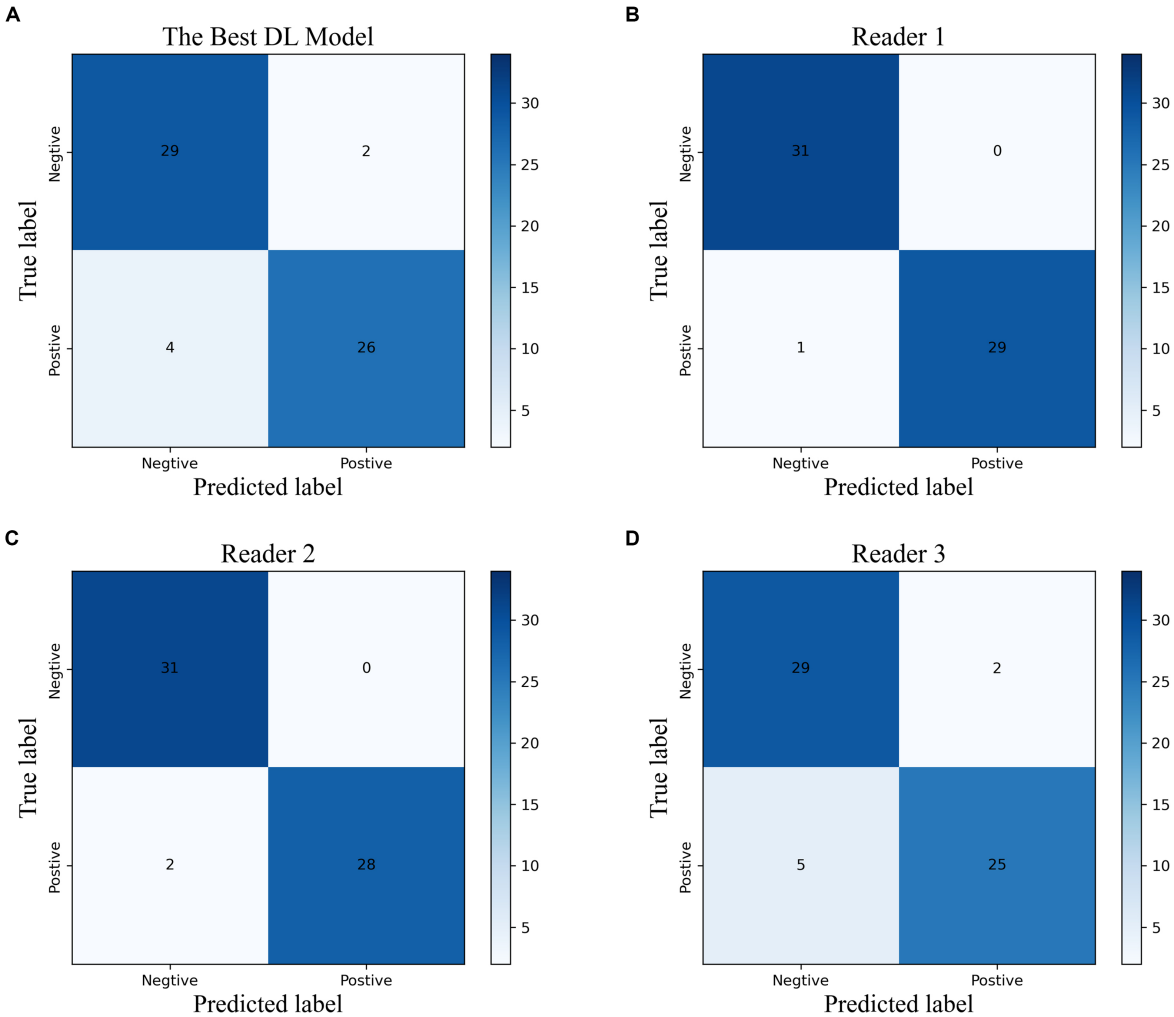
The confusion matrix of the best DL model **(A)** and the radiologists with different experiences **(B–D)** in the temporal validation cohort.

Based on the time-independent validation set, we compared the diagnostic efficacy of the best DL model with that of radiologists with different levels of experiences (Figure 4; Supplementary Table S2). The best DL model outperformed Reader 3 for accuracy (0.902, 0.885, respectively), sensitivity (0.867, 0.833, respectively), specificity (0.935, 0.935, respectively), PPV (0.929, 0.926, respectively), NPV (0.879, 0.853, respectively), and F1 score (0.901, 0.885, respectively). Moreover, the performance of the best DL model was almost identical to Reader 1 (k = 0.84) and had strong inter-rater agreement with Reader 2 and Reader 3 (k = 0.80, 0.70, respectively).

We evaluated the diagnostic accuracy of the top-performing DL model and radiologists with varying levels of experience (Figure 4; Supplementary Table S2) using a time-independent validation set. Compared to Reader 3, the DL model demonstrated superior performance across all measures, including accuracy (0.902 vs. 0.885), sensitivity (0.867 vs. 0.833), specificity (0.935 vs. 0.935), PPV (0.929 vs. 0.926), NPV (0.879 vs. 0.853), and F1 score (0.901 vs. 0.885). The DL model also showed strong agreement with Reader 1 (k = 0.84) and had high inter-rater agreement with Reader 2 and Reader 3 (k = 0.80 and 0.70, respectively).

## Discussion

In this study, we developed DL models based on dynamic 4D-CTA to detect LVO and investigated the efficacy of two different input methods, namely combining and superimposing multi-phase MIP images. Our results showed that the DL algorithm had high efficacy in LVO detection, with the P1 + P2 model in the combination input approach having the highest detection efficacy. The combination of arterial and arterial–venous phase images provided richer temporal information, enabling more accurate and timely detection of LVO.

The importance of timely treatment in managing AIS is underscored by the concept of “time is brain,” emphasizing the critical role of rapid reperfusion therapy in salvaging at-risk brain tissue and improving patient outcomes (Goyal et al., 2016; Jansen et al., 2018). Society of Neurointerventional Surgery suggest an ideal time for CTA interpretation < 10 min (Smith et al., 2018). However, challenges in the emergency setting, such as the large number of patients and the shortage of neuroradiologists, may hinder meeting this time target, resulting in delays in evaluation for LVO patients. DL-based algorithms provide a solution to simplify the diagnostic process by automatically identifying LVO, thereby ensuring timely access to diagnostic imaging and intervention. Based on the strong performance of HRNet in image classification and segmentation (Ding et al., 2021; Li et al., 2021), we improved the original HRNet network for constructing our LVO DL-model The improved HRNet network excels at extracting richer semantic features, resulting in improved performance and increased utility for real-world applications.

DL has shown promise in developing models and software for diagnosing LVO (Rava et al., 2021; Yahav-Dovrat et al., 2021; Cimflova et al., 2022; Czap et al., 2022; Seker et al., 2022), mostly based on monophasic CTA. However, 4D-CTA provides dynamic multi-phase scanning, offering temporal resolution for a comprehensive assessment of hemodynamic changes in AIS patients (Frölich et al., 2014; Kortman et al., 2015). Currently, only a few DL studies have been based on 4D CTA for LVO detection. Meijs et al. (2020) used nTTS (time to signal) maps for LVO detection, while Bathla et al. (2022) employed 5 time series data for LVO detection and localization. The results of these studies demonstrated the feasibility of DL models based on 4D CTA for LVO detection, yet they did not investigate the impact of different phases.

Without increasing the radiation dose, we obtained four-phase images of arterial, arterial–venous, venous, and late venous using TDC, ensuring scientific accuracy and precision. Our results found that arterial–venous phase was comparable to arterial phase for LVO assessment, and the combination of arterial–venous and arterial phase improved performance to a greater extent, compared to the arterial phase alone. Additionally, combining the late venous phase with the arterial phase also improved LVO detection effectiveness to some extent, consistent with the results of Stib et al. (2020), who reported that the delayed phase improved the diagnostic performance of DL models. These findings suggested that vascular flow changes and pathophysiological alterations in various periods aid in LVO diagnosis, including the disappearance or invisibility of early occluded arteries, delayed enhancement of occluded arteries and collateral vessel filling (Frölich et al., 2012). However, there was no statistically significant difference in the improvement of the AUC of the multiphase joint model in our study, showing only a trend, which may be related to the relatively limited sample size.

Moreover, we investigated two input methods for DL model development: combining and superimposing MIP maps of different phases. Our results showed that the combined input method outperformed the superimposed input method, which may be attributed to the MIP technique. 4D-CTA highlights blood flow in different vessels at different phases (reflected as different brightness of the image), while MIP projects the maximum value in the same location and sequence onto the final image. With the combined input method, by combining and stitching separate MIP images from each period, vascular changes can be observed more clearly. Conversely, the superimposed input method projects highlighted areas from each stage onto the final image, leading to reduced image quality and negatively impacting the final performance of the DL model. It underscoring the importance of selecting appropriate input methods in DL model development.

Currently, although all CTAs are reviewed by radiologists, there is no guarantee that LVO will be prioritized among the many suspected AIS cases. The comparative analysis between the best DL model and radiologists with varying levels of expertise revealed good consistency. The best DL model demonstrated a relatively higher accuracy and sensitivity, comparable specificity to less experienced trainee radiologist. These findings further emphasize the potential of DL models as valuable diagnostic support tools in prioritizing LVO detection and facilitating timely treatment decisions. It can flag the presence of LVO in unreviewed reports, alerting radiologists for prioritized review and preventing delayed diagnosis or omission.

Additionally, NIHSS scores showed a significant difference between the LVO and non-LVO groups, highlighting its role as a major clinical predictor of LVO (Fischer et al., 2005; Heldner et al., 2013) and its current utility in prehospital EVT decision-making (Václavík et al., 2018; Cabal et al., 2021). However, NIHSS assessments are relatively time-consuming and dependent on experienced neurologists. In contrast, the best DL model outperformed the NIHSS by visually detecting occluded vessels using comprehensive information from CTA images, offering more effective guidance for treatment decisions.

Our study had several limitations. Firstly, this was a single-center study with a relatively small sample size and CTA images obtained from the same scanner with consistent acquisition protocols, leading to potential sample selection bias. Future studies should consider multicenter studies with larger sample sizes and images from different acquisition parameters. Secondly, the proposed DL algorithm was not externally validated using a validation set from another institution, but a temporal validation set was used to assess its performance over time. The generalization capability of the algorithm needs to be further evaluated on multicenter external data. Thirdly, the algorithm only identifies LVO of the ICA, M1, and M2, excluding posterior circulation LVO, and does not provide information about a specific occluded segment. Therefore, we will collect more LVO cases in the anterior and posterior circulation and enhance the algorithm to better aid radiologists in detecting LVO.

## Conclusion

In conclusion, our study highlights the potential of the DL model in detecting LVO in AIS patients using 4D-CTA. Through the utilization of advanced image processing techniques and comprehensive temporal information, DL models can accurately and rapidly identify LVO, especially when using the combination of arteriovenous phase images and arterial phase images, which demonstrated the highest detection efficacy. This helps to accelerate the screening and diagnosis of LVO in urgent clinical settings, ultimately improving patient prognosis.

## Data availability statement

The raw data supporting the conclusions of this article will be made available by the authors, without undue reservation.

## Ethics statement

The studies involving humans were approved by the First Affiliated Hospital of Chongqing Medical University. The studies were conducted in accordance with the local legislation and institutional requirements. The participants provided their written informed consent to participate in this study.

## Author contributions

YP: Conceptualization, Data curation, Formal analysis, Investigation, Methodology, Writing – original draft, Writing – review & editing. JLiu: Data curation, Investigation, Validation, Writing – review & editing. RY: Formal analysis, Methodology, Software, Visualization, Writing – review & editing. JWu: Data curation, Investigation, Writing – review & editing. JLi: Methodology, Writing – review & editing. LD: Validation, Writing – review & editing. SG: Data curation, Validation, Writing – review & editing. YY: Data curation, Validation, Writing – review & editing. YL: Supervision, Writing – review & editing, Resources. SC: Project administration, Resources, Software, Supervision, Writing – review & editing. JWa: Conceptualization, Methodology, Project administration, Resources, Supervision, Writing – review & editing.
