# Supplementary material for: Deep learning-assisted diagnosis of large vessel occlusion in acute ischemic stroke based on four-dimensional computed tomography angiography
Source: Front Neurosci. 2024 Apr 10;18:1329718. doi: 10.3389/fnins.2024.1329718 (PMC11039833; doi:10.3389/fnins.2024.1329718)
Supplement: Supplementary file 1 [file Data_Sheet_1.docx]

| Single phase |  | *p* Value (reference, P1) | Combination of different phase |  | *p* Value (reference, P1) | Superimposition of different phase |  | *p* Value (reference, P1) |
| --- | --- | --- | --- | --- | --- | --- | --- | --- |
| P1 vs P2 | Internal validation set | 0.499 | P1+P2 | Internal validation set | 0.055 | MIP (P1, P2) | Internal validation set | 0.823 |
|  | temporal validation set | 0.936 |  | temporal validation set | 0.063 |  | temporal validation set | 0.955 |
| P1 vs P3 | Internal validation set | 0.336 | P1+P3 | Internal validation set | 0.978 | MIP (P1, P3) | Internal validation set | 0.695 |
|  | temporal validation set | 0.077 |  | temporal validation set | 0.294 |  | temporal validation set | 0.063 |
| P1 vs P4 | Internal validation set | 0.423 | P1+P4 | Internal validation set | 0.574 | MIP (P1, P4) | Internal validation set | 0.697 |
|  | temporal validation set | 0.001 |  | temporal validation set | 0.030 |  | temporal validation set | 0.098 |
|  |  |  | P2+P3 | Internal validation set | 0.806 | MIP (P2, P3) | Internal validation set | 0.979 |
|  |  |  |  | temporal validation set | 0.268 |  | temporal validation set | 0.271 |
|  |  |  | P2+P4 | Internal validation set | 0.627 | MIP (P2, P4) | Internal validation set | 0.075 |
|  |  |  |  | temporal validation set | 0.397 |  | temporal validation set | 0.013 |
|  |  |  | P3+P4 | Internal validation set | 0.645 | MIP (P3, P4) | Internal validation set | 0.300 |
|  |  |  |  | temporal validation set | 0.154 |  | temporal validation set | 0.018 |
|  |  |  | P1+P2+P3 | Internal validation set | 0.353 | MIP (P1, P2, P3) | Internal validation set | 0.644 |
|  |  |  |  | temporal validation set | 0.122 |  | temporal validation set | 0.092 |
|  |  |  | P1+P2+P4 | Internal validation set | 0.889 | MIP (P1, P2, P4) | Internal validation set | 0.591 |
|  |  |  |  | temporal validation set | 0.575 |  | temporal validation set | 0.433 |
|  |  |  | P1+P3+P4 | Internal validation set | 0.618 | MIP (P1, P3, P4) | Internal validation set | 0.063 |
|  |  |  |  | temporal validation set | 0.774 |  | temporal validation set | 0.402 |
|  |  |  | P2+P3+P4 | Internal validation set | 0.767 | MIP (P2, P3, P4) | Internal validation set | 0.419 |
|  |  |  |  | temporal validation set | 0.787 |  | temporal validation set | 0.264 |
|  |  |  | P1+P2+P3+P4 | Internal validation set | 0.466 | MIP (P1, P2, P3, P4) | Internal validation set | 0.679 |
|  |  |  |  | temporal validation set | 0.070 |  | temporal validation set | 0.048 |

Table S1 The Delong test results among the DL models

Table S2 The comparison of performance between the best DL model P1＋P2 and three radiologists with different experience in the time-independent validation set

|  |  | Radiologists | | |
| --- | --- | --- | --- | --- |
|  | The best DL model | Reader 1 | Reader 2 | Reader 3 |
| Accuracy | 0.902 | 0.984 | 0.967 | 0.885 |
| Sensitivity | 0.867 | 0.967 | 0.933 | 0.833 |
| Specificity | 0.935 | 1.000 | 1.000 | 0.935 |
| PPV | 0.929 | 1.000 | 1.000 | 0.926 |
| NPV | 0.879 | 0.969 | 0.939 | 0.853 |
| F1 score | 0.901 | 0.984 | 0.967 | 0.885 |
